# Practical Pathology of the Mucous Membrane of the Nose, Throat, and Ears of Children

**Published:** 1896-06

**Authors:** Thomas F. Rumbold

**Affiliations:** St. Louis, Mo.


ATLANTA
Medical and Surgical Journal.

VOL. XIII. JUNE, 1896. No. 4.

LUTHER B. GRANDY, M.D., MANAGING EDITOR.

M. B. HUTCHINS, M.D., BUSINESS MANAGER.

COLLABORATORS:
H. V. M. MILLER, M.D., LL.D., A. W. CALHOUN, M.D., LL.D., VIRGIL O. HARDON, M.D., FLOYD W. McRAE, M.D., AND DUNBAR ROY, M.D.

### ORIGINAL COMMUNICATIONS.

### PRACTICAL PATHOLOGY OF THE MUCOUS MEMBRANE OF THE NOSE, THROAT, AND EARS OF CHILDREN.

NUMBER II.

BY THOS. F. RUMBOLD, M.D.,
ST. LOUIS, MO.

In the previous paper (page 145) the abnormal conditions of the nose, throat, and ears of infants were considered; in this article the abnormal conditions of the same organs of children will be described. It was not the intention in the last paper, nor is it in this one, or in the ones following on this subject, to describe all the pathological conditions that might occur as a sequence of an inflammation of the organs named, but to describe the conditions as seen in the very great majority of cases within the limits of the ages named. Because of the great number of this kind of cases it is possible and profitable to classify them, they all being of medium severity. Cases of excessive severity, or those of an unusual kind, cannot be classified, there not being enough of them that assume the same degree of severity; they must be described singly.

*The Nasal Passages.* The inflamed nasal mucous membrane of children—from the third to the tenth year of age—differs from that of infants in the *age* of the inflammation, in the *degree* of abnormality, and in the *character* of the secretion.

A small portion of an infected nasal mucous membrane, from the inferior turbinate of a child, examined by a fifty or seventy-five objective, plainly shows that the inflammation is nearly as *old as the child.* When compared with that of an infant the difference is very manifest. The blood-vessels, the mucous glands, and the other tissues that make up the part under inspection, all give evidence of diseased action that has been for years in active progress. This shows the *degree* of abnormality. Even if we did not have daily evidence of the changed *character* of the secretions, the view under the microscope would assert this change.

While almost all portions of the nasal cavities are in some degree inflamed, the inferior turbinate processes show the greatest effect of the disease, for the reason that the nasal acrid secretion from the superior and middle turbinates has been deposited here. Wherever this irritating secretion rests there is always an increase of inflammation, an increase of diseased action. If this secretion was held on the back of one's hand it would there occasion an inflammation. This inflammation would soon be followed by swelling, and ultimately this swelling would become permanent, that is, it would become a growth. This growth, if in the nasal cavity, would decrease the normal air-space, so that there would be an impediment to the entrance of air to the lungs. The effort to maintain the normal amount of air in the lungs would produce an air-pump effect on all blood-vessels in all parts posterior to the air-obstructing growth. This air-exhaustion, be it ever so slight, is *sure* not only to maintain but to increase congestion, showing that the disease is now self-sustaining.

*The Turbinate Processes.* While the inflammation commences in the superior portion of the nasal cavity, that is where the stream of inspired air strikes the mucous membrane most forcibly, yet the diseased action is seen to be greater in the lower portions of the cavity. This is owing, as stated above, to the irritating effect of the acrid nasal secretion. The longer this secretion is exposed to the air the more acrid it becomes. By the time it reaches the inferior turbinate process it is far more active in producing abnormal conditions than is the primary disease, showing the great importance of protecting these portions of the nasal cavities from the presence of this acrid secretion.

When the turbinates are examined, post mortem, it is often seen that the blood-vessels of the inferior turbinates are quite large, from four to ten diameters greater than normal vessels. The arteries and veins are still distinguishable; this fact is an evidence that the size of the vessels does not preclude their return to the normal state. When blood-vessels lose their arterial and venous characteristics, as they do in some of the older grades, I do not think that they will return to the normal condition. The middle turbinate shows but slight abnormal change, and the superior turbinate seldom appears abnormal, again plainly showing that the acrid nasal secretion does far greater injury to the mucous membrane, by its retention on the surface, than is done by the primary irritating cause itself—that is, the colds.

*Mouth-Breathing.* If the disease has been going on unabated with but slight increased severity since infancy, normal respiration will be interfered with, the breathing space through the nasal passages will be lessened either by swellings or growths. In this condition respiration through the mouth *must* take place. It is dangerous to say to a patient that this mouth-breathing is a habit, if it is dangerous to misguide a patient in an ailment. If you say to a boy or a girl, "breathe through your nose," he or she may be able to do so for some time, but if it requires a constant effort, the mouth will open again. There is not any doubt that the effect of forcing cool air through the heated nostrils is beneficial to the swollen mucous membrane, but every person that requires this forced respiration should be a patient of a rhinologist, for the disease will soon compel him to breathe through the mouth whether he will or no, for mouth-breathing induces greater congestion, and additional swellings and growths.

*Epistaxis* is not an uncommon consequence of the excessive engorgement of the blood-vessels, and is an evidence of such rapid dilation of the vessels that their walls are ruptured. Of course all nose-bleeders have chronic nasal inflammation of more or less severity. If the nervous system does not receive a shock from a protracted nose-bleed, the hemorrhage will have a relieving effect, as the congestion that caused the rupture of the blood-vessels is usually severe enough to occasion disagreeable head symptoms that the loss of the blood relieves.

*Adenoid Growths.* In infancy there are usually no sphenoid cells, and the posterior ethmoid cells of each side are only large enough to hold about two or three drops of water. At the age of from four to six years—the age when adenoid growths appear—these cells have from four to five times greater capacity and the sphenoid cells will hold about five to eight drops of water. It is mostly the abnormal secretions from these cells—which is quite fluid and very acrid—that causes these growths. This irritating secretion leaves the cells and enters the nasal cavities by openings under the superior turbinate processes. The tendency of the secretion is to pass down the posterior portion of the upper surface of the nasal cavities. It then glides along the roof of the pharyngo-nasal cavity until it reaches the locality where the inspired air strikes this cavity. At this place the abnormally heated air drives away so large a portion of the water of the secretion that it becomes thicker and remains adherent to the mucous membrane. The fact that this acrid secretion remains in one location gives it an excellent opportunity to irritate the mucous glands, and thus occasion the adenoid growths which sprout from these glands. If this irritating secretion had lodged in any other locality where there was a bunch of glands, it would have occasioned growths at that place also.

As soon as the secretion from the sphenoid and ethmoid cells becomes thick, which it almost always does as the child gets older, then it does not flow to the wall of the pharyngo-nasal cavity; consequently no irritating secretion flows to maintain irritation enough to sustain the adenoid growths, then the absorbents take away the growths. This accounts for their disappearance at the ages from seven to twelve years. These growths require for their sustenance a large quantity of blood to maintain them, such as the supply occasioned by the acrid secretion. Sometimes they are seen after this age. I have had a number of such cases, one that of a young man twenty-seven years of age, who had these growths developed so that they appeared below the lower edge of the soft palate.

That the adenoid growths of the pharyngo-nasal cavity are caused by acrid secretion from the sphenoid and ethmoid cells is proved by making an application of very small pledgets of cotton to the highest portion of the nasal cavities (if large pieces of cotton are used the infant cannot tolerate it). This cotton will direct the stream of secretion into the middle of the passages instead of down the posterior wall. The cotton may be allowed to remain there for two days, then removed and replaced by another piece. In two weeks at most the beneficial effect of this course will be manifest. This method of treating some cases is followed by good results.

*Ears.* In the child the inflammation is of such an age that the secretion is not so fluid as in infancy. It has more pus in it, and the heat—due to increased diseased action—is greater, so that much of the watery portion of the secretion is driven away, consequently the outflow is thicker; this, with the fact that a child does not lie on its back nearly so much as an infant, accounts for the nasal secretion not going up into the Eustachian tubes. For these reasons acute ear-troubles do not frequently originate in this grade. If a child does have earaches, it is altogether likely that they originated in infancy.

When the ears are affected, it is generally due to an inflamed condition of the mucous membrane of the Eustachia canal; the inflammation occasioning a deposit of muco-purulent secretion in the canal that results in marked deafness. This obstructing secretion can only be removed by the inflation of the middle ear and canal with air. Sometimes a fibrinous deposit takes place, then there may be a stricture of the canal, which is always quite a serious ailment, and very seldom relieved. A continuous deafness always results from such strictures, one that but little or nothing can be done to improve.

*Tonsil-Growths or Tonsilloids.* These growths are uniformly, but incorrectly, called *enlarged tonsils.* They are growths on the tonsils, not an increased size of the tonsils. Because they are formed on the tonsils, I have called them tonsil-growths or tonsilloids. If these enlargements are tonsils, then the child that has enlarged tonsils excised several times would have lost his tonsils several times. I know of a case in which the tonsils were removed seven times. Is there any other organ of the body that can be excised and then *reproduced by diseased action*, and have the excision and reproduction repeated seven times? In the excision of a tonsil-growth, the tonsil is not touched, it always remains in position. The tonsil is never in sight any more than are the lingual glands or the submaxillary glands. When there is a protuberance in sight between the pillars of the arch of the soft palate, that prominence, be it great or small, is a tonsil-growth. Sometimes it has been seen on dissection that when the tonsil-growth is very large the tonsil is pushed farther inward, away from the mouth, and appears as if it was partially atrophied, apparently on account of pressure exerted by the tonsilloids.

A transverse horizontal section of a tonsil with a large tonsilloid grown from it will plainly show—if examined by the microscope with a magnifying power of about fifty diameters—the two kinds of tissue, the tonsillar structure being quite different from the tissue of the growth. In an injected specimen the blood-vessels of the tonsils are uniform in diameter, and the arteries and veins are distinguishable, while those of the growth form a plexus of the vessels of various sizes, closely resembling the growths seen on the inferior turbinates; these arteries and veins are not distinguishable.

*Tonsillar Abscess.* From what has been said it is seen that there are no abscesses in the body of the tonsil-growths. I do not think that abscesses can form in the body of such growths; abscesses can only form in inflamed normal structure. There may be an abscess in the structures adherent to the upper portion of the tonsilloid or in a tonsil that is covered by a growth, but not in the growth itself. The abscess is mostly always, if not always, above the tonsilloid. The reason for this is that the tonsilloid grows from the tonsil and extends downward; the tonsil is always at the upper part of the tonsilloid. When an unassisted rupture of one of these abscesses takes place it is almost always in the lower part of the soft palate, over the growth, or where the anterior and posterior pillars of the arch of the soft palate meet, just outside of the growth and near the base of the uvula.

